# Fellowships, Grants, & Awards

**Published:** 2006-04

## Disease Investigation through Specialized Clinically Oriented Ventures in Environmental Research (DISCOVER) [P50]

The National Institute of Environmental Health Sciences (NIEHS) invites qualified investigators from academic institutions or nonprofit research institutions to submit an application for a Disease Investigation through Specialized Clinically Oriented Ventures in Environmental Research (DISCOVER) Center. Each DISCOVER Center will develop an overarching theme that is based on a specific environmentally influenced disease or dysfunction and will craft an interdisciplinary research approach that integrates patient-oriented or public health research with basic mechanistic studies to address disease etiology, pathogenesis, susceptibility, and/or progression. An extraordinary level of synergy, integration, and potential for advancement of environmental health sciences is expected. By fostering collaborative research these centers will increase the relevance of basic scientific discoveries in environmental health sciences to human disease, and move this knowledge into clinical and public health application to ultimately improve human health.

NIEHS recognizes that to accomplish this research agenda, two types of activities need to converge. First, the research team must focus on a particular human disease or dysfunction that is likely to be caused or influenced by environmental exposure(s). Second, a team of physician scientists, research scientists trained in the basic biomedical disciplines, and public health researchers will work collaboratively to use toxicant exposures or environmental perturbations to understand human disease. Thus, the research conducted by a DISCOVER Center will capitalize on multiple aspects of environmental health sciences research including exposure biology, environmental genetics and genomics, patient-oriented clinical research, and public health sciences, such as epidemiology, as well as computational and engineering approaches to define the functional contributions of environmental and genetic determinants in 1) assessing the risk of developing disease, 2) identifying the underlying physiological mechanisms in disease pathogenesis and progression, 3) characterizing disease phenotype, 4) understanding the environmental and endogenous factors that affect the distribution of disease in populations, and 5) applying the knowledge gained to develop therapeutic, diagnostic, prognostic, and preventative strategies.

The mission of the NIEHS is to promote research that will reduce the harmful effects of environmental exposures on human health and disease. The link between environmental agents and disease risk has been recognized for over a century. It has also been recognized that there is considerable variability in an individual’s response and ultimately an individual’s risk to developing disease as a consequence of exposure to environmental or lifestyle factors. The completion of the Human Genome Project has been heralded as the beginning of a new age of science where the resultant genetic advances will imminently lead to improvements in human health. However, our understanding of environmental health and the development of disease suggests a multifactorial process involving a complex interplay between genetic variability, external exposures (i.e., the environment), temporal vulnerability (i.e., age), and other unique host factors such as preexisting conditions, nutritional status, lifestyle choices, and social status. This leads to a daunting challenge for the environmental health sciences community to assess the risk of developing disease from exposures to toxicants/stressors, and to apply this knowledge for improving human health.

Most common chronic diseases such as cancer, cardiovascular disease, asthma, diabetes, reproductive diseases/disorders, autoimmune diseases, and neurobehavioral and neurodegenerative disorders are believed to have multiple genetic and environmental factors contributing to their observed phenotype. For example, more than 35 genes have been identified that are likely to contribute to asthma susceptibility, indicating the complex etiology and biology of this disease. Additionally, early life exposures to factors such as microbial toxins, pet dander, and environmental tobacco smoke influence the risk for asthma and related phenotypes in a genotype-specific manner, suggesting that environmental exposures are critical to understanding the underlying pathogenesis of asthma. Similarly, there is mounting evidence that neurodegenerative diseases such as Parkinson, Alzheimer, and dementia, not only are strongly influenced by genetic variables, but that environmental exposures during vulnerable periods of early life may contribute to disease etiology. Likewise, there is increasing evidence that early life exposures to environmental factors predispose individuals to developing obesity as adults. Obesity, along with insulin resistance, dyslipidemia, and hypertension are key clinical features of metabolic syndrome, a common disease associated with an increased risk of type 2 diabetes mellitus and cardiovascular disease. While a direct linkage between environmental exposures beyond lifestyle factors such as diet and nutrition has not been made to date, environmental exposures are likely contributing to the increasing prevalence of metabolic syndrome. In aggregate, these observations demonstrate the importance of environmental exposures in understanding the etiology, pathogenesis, and prognosis of many complex human diseases.

The contribution of the environmental component in the gene–environment paradigm to the etiology of human disease has been difficult to ascertain. This is partly due to the lack of precision in the methods to integrate exposure over time, the inability to characterize the attributable risk from multiple exposures (i.e., environmental toxicants, nutrition, and lifestyle choices) through life, and the lack of statistical and computational approaches to measure complex gene–environment, or gene–environment–comorbidity interactions. Ultimately this imprecision in the exposure measurement may underestimate the involvement of environmental factors in disease causation. The advent of the omics, transcriptomics, genomics, proteomics, and metabolomics, as well as nanotechnology and molecular imaging, are creating new sets of tools that hold promise to better quantify environmental exposures and assess early indicators of biological responsiveness. The incorporation of these approaches offers opportunities to better understand the underlying biology that confers variability in disease response as a consequence of exposure.

Historically, most research conducted by the NIEHS community has emphasized an assessment of the toxicologic effects of environmental agents on biological processes using animal models; in many instances these studies have not made direct linkages to human disease. Consequently, the translation of research supported by the NIEHS into improving human health has not been as rapid as desired. In the past few years, NIEHS has developed and supported new programs such as the Centers for Children's Environmental Health and Disease Prevention Research (http://www.niehs.nih.gov/translat/children/children.htm), the Centers for Population Health and Health Disparities (http://obssr.od.nih.gov/CPHHD/Index.htm), the Collaborative Centers for Parkinson's Disease Environmental Research (http://www.niehs.nih.gov/ccpder/), and the Breast Cancer and the Environment Research Centers (http://www.bcerc.org/), which are intended to provide a human disease focus for the NIEHS.

The DISCOVER Centers program is designed to further broaden the NIEHS research portfolio in a clinically focused direction, by encouraging the extramural community to identify and define the basic and applied research opportunities likely to have the most profound impact on the understanding and management of environmentally influenced disease. The NIEHS continues to be committed to supporting research through investigator-initiated, single-laboratory project grants that focus on the adverse effects that environmental toxicants/stressors have on cellular and biological processes. However, the resources required to conduct the multifaceted, interdisciplinary projects necessary to achieve significant advances in the characterization of human disease, and accelerate research findings into clinical and public practices that impact disease outcome are beyond the scope of a typical R01 grant. Therefore, the DISCOVER Center program is being initiated to create an integrated research approach involving teams of scientists that represent clinical research, basic biomedical research disciplines, and public health sciences. These teams will focus on complex human diseases or disorders where there is evidence or a strong rationale for the involvement of environmental factors in its etiology, phenotypic expression, or population distribution. As part of the design for these centers, the research should lead to improved clinical and public health practices through advances in prevention, diagnosis and treatment.

Each DISCOVER Center will develop an overarching theme that is based on a specific environmentally influenced disease or dysfunction and will craft an interdisciplinary research approach that incorporates clinical and basic mechanistic studies to address disease etiology, pathogenesis, susceptibility, progression, and/or prognosis. An extraordinary level of synergy and potential for advancement of environmental health sciences is expected through the creation of research projects that individually are scientifically meritorious but together display a high degree of complementarity and integration. Ultimately, these centers are expected to accelerate the application of knowledge derived from basic research into the clinical or public health setting with the goal of improving human health. The DISCOVER program is expected to create opportunities to develop and apply novel approaches for the diagnosis, prognosis, prevention, and treatment/intervention of environmentally influenced diseases or disorders.

The general characteristics of a DISCOVER Center to promote the goals of this initiative include: 1) a central theme that identifies a specific human disease or dysfunction to be studied, and well-articulated hypotheses that promote collaboration and integration between projects; 2) a series of collaborative research projects that work at the interface between environmental exposures, basic biology, genomics/genetics, clinical sciences, and population-based studies and incorporate biological model systems such as patient cohorts, genetically manipulated mouse strains, or human or animal cell lines; 3) an experimental approach that, in part, incorporates the concepts of environmental health sciences to understand human disease by employing environmental toxicants or stressors to elucidate how genes affect normal cellular or pathophysiological processes, assess how genetic variation contributes to individual susceptibility and the phenotypic diversity of human disease, or explain interindividual variability in etiology, pathogenesis, or prognosis; 4) a research plan to facilitate the extension of knowledge gained to clinical and/or public health practice.

A DISCOVER Center must reflect an integrated research enterprise that will advance our understanding of how environmental stimuli interact with biological processes to either preserve health or cause disease. Applicants may focus their center on any human disease or dysfunction provided there is adequate justification for the role of primary environmental stressors in influencing disease etiology, progression, prognosis, or population distribution. The NIEHS defines environment quite broadly, and for this initiative considers chemical, physical, or biological toxicants as primary stressors. In addition to a focus on primary stressors, applicants should investigate the effects of secondary modifiers such as comorbid disease/conditions, aging, diet, infectious disease, and/or idiosyncratic drug reactions that have the possibility to influence the susceptibility to physical or environmental toxicant exposures and thereby alter disease processes. For this initiative proposals focusing solely on secondary modifiers, including but not limited to smoking, alcohol, infectious agents, or diet, in the absence of primary exposures, will not be considered responsive.

The scientific theme developed for a DISCOVER Center should reflect an interdisciplinary research approach that incorporates multiple levels of scientific endeavors. Examples of general themes and approaches that may be considered appropriate for inclusion within a DISCOVER Center include but are not limited to the following suggestions.

Characterize disease occurrence in terms of etiologic heterogeneity, disease pathogenesis, or by identifying biological targets for understanding gene–environment interactions and novel approaches to therapeutic intervention. 1) Use environmental exposures to identify the cellular and molecular patterns of disease that characterize unique biological phenotypes and provide additional understanding of disease processes. 2) Use environmental exposure to understand basic mechanisms of disease pathogenesis. 3) Screen for molecular abnormalities that relate to particular steps or manifestations of the disease. 4) Use environmental agents as probes to dissect critical pathway components that lead to, modulate, or augment the progression or risk of disease. These environmental probes can help develop novel therapeutic/diagnostic tools. 5) Employ comparative biology and functional genomic, proteomic, or metabolomic studies across multiple species in parallel with human studies in the presence of environmental agents to facilitate the discovery of basic underlying mechanisms of disease.

Clarify the contribution of environmental and genetic variables in the risk of developing disease and the risk of disease progression. 1) Assess the clinical implications of cellular and molecular patterns for disease susceptibility, development, or progression and therapeutic intervention. 2) Investigate the interactions between comorbid conditions on individual sensitivity to environmental factors in the disease process. 3) Conduct studies in well characterized exposed populations to identify factors that lead to variation in an individual’s susceptibility to disease. These markers of disease susceptibility could then be further validated within a clinical research environment to assess the functional relevance of gene–environment interactions as it contributes to the development and progression of human disease.

Stratify disease risk and target intervention to promote improved health at the individual or population level. 1) Conduct studies in humans that may have direct diagnostic or therapeutic application, e.g., genetic polymorphisms that relate to disease susceptibility, clinical outcome, or assessing the clinical effectiveness of a therapeutic intervention. 2) Identify molecular and cellular targets for novel therapies to mitigate the effects of environmental exposures. 3) Apply omics, nanotechnology, and imaging approaches across species in parallel with human studies to develop biomarkers of exposure, disease disposition, onset, severity, or progression. 4) Use environmental agents as tools to understand perturbations in normal cell physiology to directly link exposure to disease. This could lead to the identification and validation of biomarkers and their application as prognostic indicators of human diseases as well as therapeutic efficacy.

A DISCOVER Center must also have a strong focus on translational research which has been defined by NIH as studies at the interface of the bench and bedside and/or community. Information flow at these interfaces is bidirectional, requiring close interaction between clinical and bench scientists to study human diseases. The structure of the center should facilitate the acceleration of basic research findings into practical applications that benefits clinical and public health practice to improve human health. Examples include: 1) the development of sensitive preclinical markers of exposure and biological responses; 2) the development of novel therapeutic agents and/or diagnostic tools; 3) the development of biomarkers for disease phenotype, onset, severity, or progression; 4) the application of biomarkers as prognostic indicators of human diseases as well as therapeutic efficacy.

The DISCOVER Center supports a full range of basic, developmental, clinical, public health, and/or applied research components; and is intended to result in novel scientific concepts about the etiology and pathogenesis, and distribution of human diseases. The ultimate goal of the DISCOVER Centers is to use scientific methods that are unique to environmental health sciences to further understand human health and disease.

The DISCOVER Center must have an identifiable organizational unit within a university, medical school, nonprofit research institute, or a consortium of cooperating institutions with a university affiliation. The applicant institution must include a minimum of 50% of the research effort. Partnerships may consist of investigators at a single institution or at multiple sites and may include collaborative arrangements as appropriate with organizations, domestic or foreign, public or private (such as universities, colleges, hospitals, laboratories, for-profit and nonprofit, units of state and local governments, and eligible agencies of the federal government), as necessary to conduct portions of the research. Teams that are geographically distributed must be well justified, and steps to minimize the effects of geography should be clearly stated.

The DISCOVER P50 grant mechanism fosters collaborative and integrated basic, clinical, and public health research and provides funds to support personnel, stipends and tuition for trainees, equipment, supplies, and services for research projects, facility cores, and an administrative core.

The director for the DISCOVER Center is the designated leader and provides the leadership for the administrative, scientific, and programmatic direction. It is expected that the director will commit a minimum of 15% effort to the administration of the center. Likewise, the center must reflect an emphasis on clinical research and integration by the identification of a lead physician-scientist whose role is to ensure communication and translation across the research projects by devoting a minimum of 15% effort to center administration. The physician-scientist can serve as either director or associate director of the center.

The research projects provide the intellectual and scientific direction for the program. These projects should be well integrated and relate to the theme of the center. A minimum of four research projects are required and must include at least two patient-oriented clinical/public health research projects and two mechanistically driven basic research project. Additional projects are encouraged. Project leaders are expected to devote at least 20% effort to these projects.

A required Administrative Core oversees the organizational, budgeting, and reporting aspects of the DISCOVER Center and provides the environment and infrastructure to promote cross-discipline interactions among all projects and cores. To aid the center director in achieving the goals of the program, the establishment of an external advisory committee is required. The composition of the external advisory committee should reflect the scientific expertise of the center and must include physician-scientists. The Administrative Core will also include a plan to support product development to facilitate the translation of knowledge, resources, and tools resulting from the research effort to improve human health.

Facility Cores are designed principally as a service or resource component to the research projects within the center. Core facilities may include clinical research support, biostatistics and/or bioinformatics support, and basic molecular/cellular capabilities. These cores serve to enhance or make more cost-effective the services, techniques, or instrumentation used by the center and also promotes interdisciplinary activities. The DISCOVER Center should include facility cores that serve at least two research projects if resources are not available through other Institutional infrastructure such as P30 Core Centers or other resources such as GCRCs or Clinical and Translational Science Award Centers (CTSAs). The number of facility/service cores may not exceed the total number of research projects and are not a requirement of the Discover Centers. Applicants must include a discussion of the cost savings and other efficiencies provided by the inclusion of such core facilities.

This funding opportunity will use the P50 award mechanism. As an applicant, you will be solely responsible for planning, directing, and executing the proposed project.

This funding opportunity uses the just-in-time budget concepts. It also uses the nonmodular budget format described in the PHS 398 application instructions (see http://grants.nih.gov/grants/funding/phs398/phs398.html). A detailed categorical budget for the Initial Budget Period and the Entire Proposed Period of Support is to be submitted with the application.

The PHS 398 application instructions are available at http://grants.nih.gov/grants/funding/phs398/phs398.html in an interactive format.

Applicants must use the currently approved version of the PHS 398. For further assistance contact GrantsInfo, 301-435-0714 (telecommunications for the hearing impaired: TTY 301-451-0088) or by e-mail: GrantsInfo@nih.gov. Applications must be prepared using the most current PHS 398 research grant application instructions and forms. Applications must have a D&B Data Universal Numbering System (DUNS) number as the universal identifier when applying for Federal grants or cooperative agreements. The D&B number can be obtained by calling 866-705-5711 or through the web site at http://www.dnb.com/us/. The D&B number should be entered on line 11 of the face page of the PHS 398 form.

The letter of intent receipt date for this RFA is October 17, 2006, with the application receipt date November 17, 2006. The complete version of the RFA is available at http://grants.nih.gov/grants/guide/rfa-files/RFA-ES-06-001.html.

Contact: David M. Balshaw, Center for Risk and Integrated Sciences, Division of Extramural Research and Training, National Institute of Environmental Health Sciences, PO Box 12233, EC-27, 79 T.W. Alexander Drive, Research Triangle Park, NC 27709 USA, 919-541-2448, fax: 919-541-4937, e-mail: Balshaw@niehs.nih.gov; Kimberly Gray, Susceptibility and Population Health Branch, Division on Extramural Research and Training, National Institute of Environmental Health Sciences, PO Box 12233, EC-21, 79 T.W. Alexander Drive, Research Triangle Park, NC 27709 USA, 919-541-0293, fax: 919-316-4606, e-mail: gray6@niehs.nih.gov; Jerry Heindel, Cellular, Organ and Systems Pathobiology Branch, Division on Extramural Research and Training, National Institute of Environmental Health Sciences, PO Box 12233, EC-23, 79 T.W. Alexander Drive, Research Triangle Park, NC 27709 USA, 919-541-0781, fax: 919-541-5064, e-mail: heindelj@niehs.nih.gov. Reference RFA-ES-06-001

